# How should ICU beds be allocated during a crisis? Evidence from the COVID-19 pandemic

**DOI:** 10.1371/journal.pone.0270996

**Published:** 2022-08-10

**Authors:** Charlotte M. Dieteren, Merel A. J. van Hulsen, Kirsten I. M. Rohde, Job van Exel

**Affiliations:** 1 Erasmus School of Health Policy & Management, Erasmus University Rotterdam, Rotterdam, The Netherlands; 2 Erasmus Centre for Health Economics Rotterdam (EsCHER), Rotterdam, The Netherlands; 3 Erasmus School of Economics, Erasmus University Rotterdam, Rotterdam, The Netherlands; 4 Erasmus Research Institute of Management, Erasmus University Rotterdam, Rotterdam, The Netherlands; 5 Tinbergen Institute, Erasmus University Rotterdam, Rotterdam, The Netherlands; Duke University, UNITED STATES

## Abstract

**Background:**

The first wave of the COVID-19 pandemic overwhelmed healthcare systems in many countries, and the rapid spread of the virus and the acute course of the disease resulted in a shortage of intensive care unit (ICU) beds. We studied preferences of the public in the Netherlands regarding the allocation of ICU beds during a health crisis.

**Methods:**

We distributed a cross-sectional online survey at the end of March 2020 to a representative sample of the adult population in the Netherlands. We collected preferences regarding the allocation of ICU beds, both in terms of who should be involved in the decision-making and which rationing criteria should be considered. We conducted Probit regression analyses to investigate associations between these preferences and several characteristics and opinions of the respondents.

**Results:**

A total of 1,019 respondents returned a completed survey. The majority favored having physicians (55%) and/or expert committees (51%) play a role in the allocation of ICU beds and approximately one-fifth did not favor any of the proposed decision-makers. Respondents preferred to assign higher priority to vulnerable patients and patients who have the best prospect of full recovery. They also preferred that personal characteristics, including age, play no role.

**Conclusion:**

“Our findings show that current guidelines for allocating ICU beds that include age as an independent criterion may not be consistent with societal preferences. Age may only play a role indirectly, in relation to the vulnerability of patients and their prospect of full recovery. Allocation of ICU beds during a health crisis requires a multivalue ethical framework.”

## Introduction

During the first quarter of 2020, there were more than 118,000 confirmed cases of COVID-19 in at least 114 countries [1]. Consequently, the WHO officially declared the international COVID-19 outbreak a pandemic in March, 2020 [1]. The first wave of the COVID-19 pandemic overwhelmed healthcare systems in many of these countries [2–4]. The acute course of the disease, which includes respiratory conditions that sometimes require admission to an intensive care unit (ICU), revealed that even some of the better equipped healthcare systems faced a shortage of ICU beds.

Resource scarcity in healthcare is not a new phenomenon. In most countries, the demand for healthcare exceeds the capacity for delivery within the available budget. As a consequence, choices must be made about how to spend these resources optimally. Although countries are thus familiar with rationing scarce health care resources, the scarcity due to the COVID-19 outbreak had a different character. The pandemic led to situations of acute shortages of both medical devices, such as high-filtration N-95 masks and ventilators, and specialized staff. Italy, the European epicenter of the COVID-19 pandemic in February 2020, initially faced an extreme shortage of ICU beds and staff, which forced physicians to allocate critical resources to the patients who would benefit most [5]. Italian physicians were supported with recommendations by an Italian expert team (SIAARTI) on how to prioritize patients in times of ICU bed scarcity [6]. The criteria to be considered for admitting patients to ICUs included age, comorbidities, and pre-existing functional status. However, these recommendations were strongly criticized by the media and the public as ageist and discriminatory against elderly patients [7]. Other countries had similar experiences during the first wave [8].

Decisions about who to treat and who not to treat sometimes lead to intense societal and political debates. Aligning decisions with societal preferences may help increase public acceptance and support for such decisions. However, previous research has shown that societal preferences are heterogeneous; along with broader ethical notions such as fairness, solidarity, and equity, members of the public care about the effectiveness of the treatment, the severity of the disease, patients’ capacity to benefit, and the size of the gains in terms of quality of life [9–11]. Moreover, such societal preferences may well be different in crisis situations where the consequences of allocation decisions are more salient because they affect more people and are more ambiguous due to uncertainties about the nature of the crisis. It is well known from research in psychology and behavioral economics that salience and ambiguity affect people’s preferences [12–14]. However, it is unclear whether these societal preferences are affected by crisis situations that strongly impact healthcare systems, such as catastrophes and epidemics. Catastrophes (e.g., natural disasters, airplane crashes) mostly have courses that are easier to predict than those of epidemics (e.g., Ebola, SARS). In addition, the magnitudes of pandemics like the COVID-19 outbreak place such heavy burdens on healthcare systems that they also affect other patients. For instance, the capacity for regular care must be scaled down and the treatment of other patients displaced [15,16]. The Netherlands’ National Institute for Public Health and the Environment estimated that, during the country’s first wave alone, at least 50,000 healthy years of life were lost as a consequence of delayed or cancelled appointments with medical specialists [17]. Hence, rationing during a pandemic requires careful understanding of the overall situation.

The current COVID-19 pandemic makes it possible to investigate public support for rationing decisions made during a health crisis. During the first wave of the pandemic, by the end of March 2020, hospitals in the Netherlands experienced a critical shortage of medical equipment and staff, and in some regions of the country a shortage of ICU capacity was imminent [18]. Experts and healthcare professionals in several hospitals raised concerns about the escalating situation, some patients were deferred to hospitals in other parts of the country and also to Germany, and the national government commissioned the development of guidelines for prioritizing patients in need of intensive care [18]. The public debate about this crisis situation in the healthcare system and about the need for prioritizing among patients also intensified. The present study aimed to investigate preferences among the public in the Netherlands regarding allocation of ICU beds in times of healthcare crises, looking both at who should be involved in the decision-making and which rationing criteria should be considered. In addition, we explored the relation between these preferences and respondents’ demographic characteristics as well as their opinions about the government’s response to the pandemic. These additional analyses provide more insight into the heterogeneity of the measured preferences and will help identify the groups in a society who will potentially support or oppose different policies or guidelines proposed by different stakeholders. As a consequence, allocation guidelines can be aligned with societal preferences, which will increase the policy acceptance rate among the public. In addition, information about the heterogeneity within the public can also be used to more effectively inform the public about why such allocation policies are needed.

## Methods

### Survey design and sample

In this study, we used data collected at the end of March 2020 to investigate the compliance of citizens in the Netherlands with government measures to contain and mitigate the spread of the coronavirus [19]. At the time, which was one month after the first confirmed case of COVID-19, the Netherlands experienced exponential growth in the number of infections and hospital admissions, and the imminent scarcity of ICU beds was starting to become a topic of public debate. To collect the data, we developed a survey. This survey was programmed and distributed online by a survey sampling company. Invitations (and reminders) to participate in this survey were sent via email to members of their panel. Respondents were recruited using a quota-sampling approach, which aimed for the respondents being comparable to the Netherlands’ adult population in terms of age, sex and level of education. The target sample size was 1,000 respondents. No formal sample size calculations were performed. At the beginning of the survey, respondents were given information about the purpose of the study and were instructed that their participation was voluntary and anonymous to the researchers and that they could end their participation at any time. The full questionnaire, translated to English by the authors, is available upon request.

### Measures

#### Who should be involved in making decisions about the allocation of ICU beds?

To assess who members of the public believe should be involved in decision-making regarding the allocation of ICU beds, we presented respondents with a list of ten decision-makers that could potentially have a role in developing guidelines for prioritizing patients for ICU beds in the Netherlands (see Table 1). This list was compiled based on the current guidelines for allocation of ICU beds in the Netherlands and discussions at the time in parliament, the medical profession and in the media about who should be involved in decision-making. Considering the topic of societal preferences in this study, we added ‘the population of the Netherlands’ to this list. We asked respondents to indicate on a 5-point Likert scale the extent to which they believed that each of these decision-makers should play a role in developing these guidelines (ranging from 1 = *completely disagree* to 5 = *completely agree*). For the analyses, we organized the decision-makers into five categories, as shown in the right-hand column of Table 1. These categories were based on the similarity in importance attached to the different types of decision-makers by respondents, by inspecting the Spearman correlations between the Likert scores (see Supporting information, S1). Despite moderate correlation, the decision-makers “population of the Netherlands” and “lottery” were placed into separate categories on substantive grounds. The decision-maker “hospital management” was not included into one of the categories and excluded from further analysis as the observed correlations did not allow for a meaningful and unambiguous classification into any one of these categories. Within the identified categories with more than one decision-maker, the Likert scores were moderately to highly correlated (i.e., between 0.47 and 0.89).

**Table 1:** Decision-makers.

*Decision-maker*		*Category*
1.	Physician on duty	Physicians
2.	Physicians from the hospital making a joint decision	
3.	National association of intensive care physicians	Expert committees
4.	Team of experts	
5.	The House of Representatives	Government
6.	The Cabinet	
7.	The Ministry of Health, Welfare and Sports	
8.	Population of the Netherlands (for instance, through a referendum)	The public
9.	Lottery (giving all patients an equal chance for an ICU bed)	Lottery
10.	Hospital management	–

The five decision-maker categories were then organized as dummy variables that take the value 1 for a respondent if the respondent’s average agreement score on the 5-point Likert scale for the decision-makers in that category was at least 4, corresponding to a ‘(completely) agree’ score that these decision-makers should play a role in developing guidelines, and the value 0 otherwise. For example, respondents who were positive that the “physician on duty” and “physicians from the hospital making a joint decision” should play a role in making allocation decisions (by giving these two potential decision-makers an average score of 4 or higher) were assigned the value 1 for the category *physicians*, while those who were negative or neutral about such a role for them (by giving them an average score of less than 4) were assigned the value 0 for this category.

Respondents were also asked whether they had additional suggestions for decision-makers that should be involved in developing the guidelines. The answers in this open text field were categorized as “no,” “don’t know,” “protest answer,” and “a specific recommendation.”

#### Allocation criteria for the rationing of ICU beds

Next, we presented respondents with a list of 18 criteria that might be considered in the development of guidelines for the allocation of ICU beds (see Table 2). These criteria were selected from previous research that has investigated societal preferences for the distribution of health and healthcare [10,20,21], combined with the most salient criteria mentioned in the public and political debates in the Netherlands at the time of the survey development. Each criterion reflects a distinct potential reason for a rationing choice. Although these criteria are not necessarily independent (e.g., age and vulnerability), we included them as separate criteria in order to try to disentangle the relevance of each criterion for priority setting in the view of the public. We asked respondents to indicate on a 5-point Likert scale whether each of these criteria should have a role in guidelines for allocating ICU beds (ranging from 1 = *completely disagree* to 5 = *completely agree*).

**Table 2:** Potential criteria for rationing ICU beds.

1. The most vulnerable patient should receive priority
2. Younger patient should receive priority
3. Patient who has been to the hospital for care before should receive priority
4. Patient who arrives at the hospital first should receive priority
5. Patient who had a higher risk of becoming infected because of working in a crucial profession during the coronavirus outbreak (such as health care, police, grocery stores) should receive priority
6. Patient who had a higher risk of becoming infected because of working on the development of a treatment against the coronavirus should receive priority
7. Patient who had a higher risk of becoming infected because of providing care to people with the coronavirus should receive priority
8. Patient with the highest chances of full recovery should receive priority
9. Patient who are breadwinners should receive priority
10. Patient who provides informal care to family members should receive priority
11. Patient who is parent of school-going children should receive priority
12. Patient who has not used much healthcare in the past should receive priority
13. Patient who was completely healthy before becoming infected should receive priority
14. Patient who complied with precautionary measures should receive priority
15. Patient with urgent needs based on a reason other than coronavirus should receive priority
16. Patient with coronavirus should receive priority
17. Patient who lives near the hospital should receive priority
18. Personal characteristics of patients should play no role in deciding who gets an ICU bed

#### Opinion variables

We also collected data on respondents’ opinions about the government’s response to the pandemic. We asked respondents whether they considered the government’s response to the pandemic to be highly insufficient, insufficient, appropriate, exaggerated, or highly exaggerated, as well as whether they believed that the measures taken by the government were very effective, effective, neutral, ineffective, or very ineffective in combating the pandemic. We also asked whether respondents had been stockpiling food and household goods, as a proxy for the experienced uncertainty about the development of the COVID-19 crisis. The rapid spread of this novel virus came with great uncertainty about its health effects and its impact on the economy and society at large. Such uncertainty in a time of crisis has been shown to affect household consumption and stockpiling [22]. The exact wording of these questions can be found in the Supporting information (S2 Table).

#### Demographic characteristics

Finally, we asked respondents about several demographic characteristics, including age, sex, employment status, and highest achieved level of education.

### Analytical approach

We only included respondents who completed the survey. After cleaning and recoding the variables of interest for this study, we examined the sociodemographic characteristics of the sample and their answers to the two central questions. We first recoded the answers to the question about who should be involved in the development of the allocation guidelines into five categories, as described above. Next, we further analyzed these answers in terms of the number of decision-maker categories respondents thought should be involved in the development of the guidelines, distinguishing in particular the group of respondents who assigned a score of 3 or less to all categories. For the question about the criteria that should be used in decision-making, we computed the mean score for each of the 18 decision-making criteria, based on the scores of respondents on the 5-point Likert scale, and their difference from the overall mean score across criteria.

Second, we estimated a series of binary response models to examine the relationship between the respondents’ preferences and their demographic characteristics and opinions. First, we examined the relationships between the demographic characteristics of respondents (i.e., age, sex, level of education, and employment) and their probability of being in favor of the involvement of each decision-maker category (i.e., physicians, expert committee, government, the public, lottery). Then we added opinions about the government’s response to the COVID-19 pandemic (i.e., whether the response was sufficient, whether the measures were effective, and whether respondents engaged in stockpiling) to these models. The following model structure was applied:

$${\text{Y}}_{\text{i}}=\left\{\begin{array}{c} 1\\ 0 \end{array}\right.\begin{array}{c} {\;\;\;\;\text{if}\;\text{X}}_{\text{i}}\theta \;+{\;\varepsilon }_{\text{i}}\;>\;0\\ \text{otherwise}\;\;\;\;\;\;\; \end{array}$$

where Y_i_ is the binary outcome variable (i.e., a decision-maker category or a decision-making criterion) and X_i_ captures a number of demographic characteristics and opinions of respondents. The parameter ε_i_ denotes the error term.

Next, we estimated models for the six decision-making criteria that were the most relevant according to the respondents or were the most heavily discussed in public and political debates in the Netherlands at the time of data collection. To do this, we first examined the relationships between the respondents’ demographic characteristics (i.e., age, sex, level of education, and employment) and the probability of being in favor of each of the six decision-making criteria. Then we added the preferences for the five decision-maker categories to the models. We used STATA 16.0 to analyze the data.

### Ethical approval

This study was approved by the Internal Review Board of the Erasmus School of Economics (ESE IRB-NE application 2020–04). Participants could only continue with the survey once they provided written informed consent.

## Results

### Study sample

Table 3 shows the demographic characteristics of the 1,019 respondents who returned completed surveys. The final column shows the percentages of the reference population during the data collection. The mean age was 48 years and 53% of the sample were female. The sample is slightly higher educated than the reference population.

**Table 3:** Demographic characteristics and COVID-19-related opinions of the study sample (*N* = 1,019) and proportions in overall population.

*Demographic characteristics*		Sample	Population^1^
		N (%)	%
Age	18–34	248 (24.3)	25.8
	35–59	474 (46.5)	45.8
	60–77	297 (29.2)	28.4
Sex	Female	542 (53.2)	51.0
	Male	477 (46.8)	49.0
Education level	Low	288 (28.3)	31.7
	Medium	370 (36.3)	37.8
	High	361 (35.4)	30.5
Employed	No	471 (46.2)	
	Yes	548 (53.8)	
*COVID-19 related opinions*			
Government response	(Highly) Insufficient	266 (26.1)	
	Appropriate	657 (64.5)	
	(Highly) Exaggerated	96 (9.4)	
Government measures	(Highly) Ineffective	132 (13.0)	
	Neutral	314 (30.8)	
	(Highly) Effective	573 (56.2)	
Stockpiling	No	687 (67.4)	
	Yes	332 (32.6)	

^1^ Quota provided by survey sampling company, based on national statistics.

### Who should be involved in decisions about the allocation of ICU beds?

**Fig 1:**
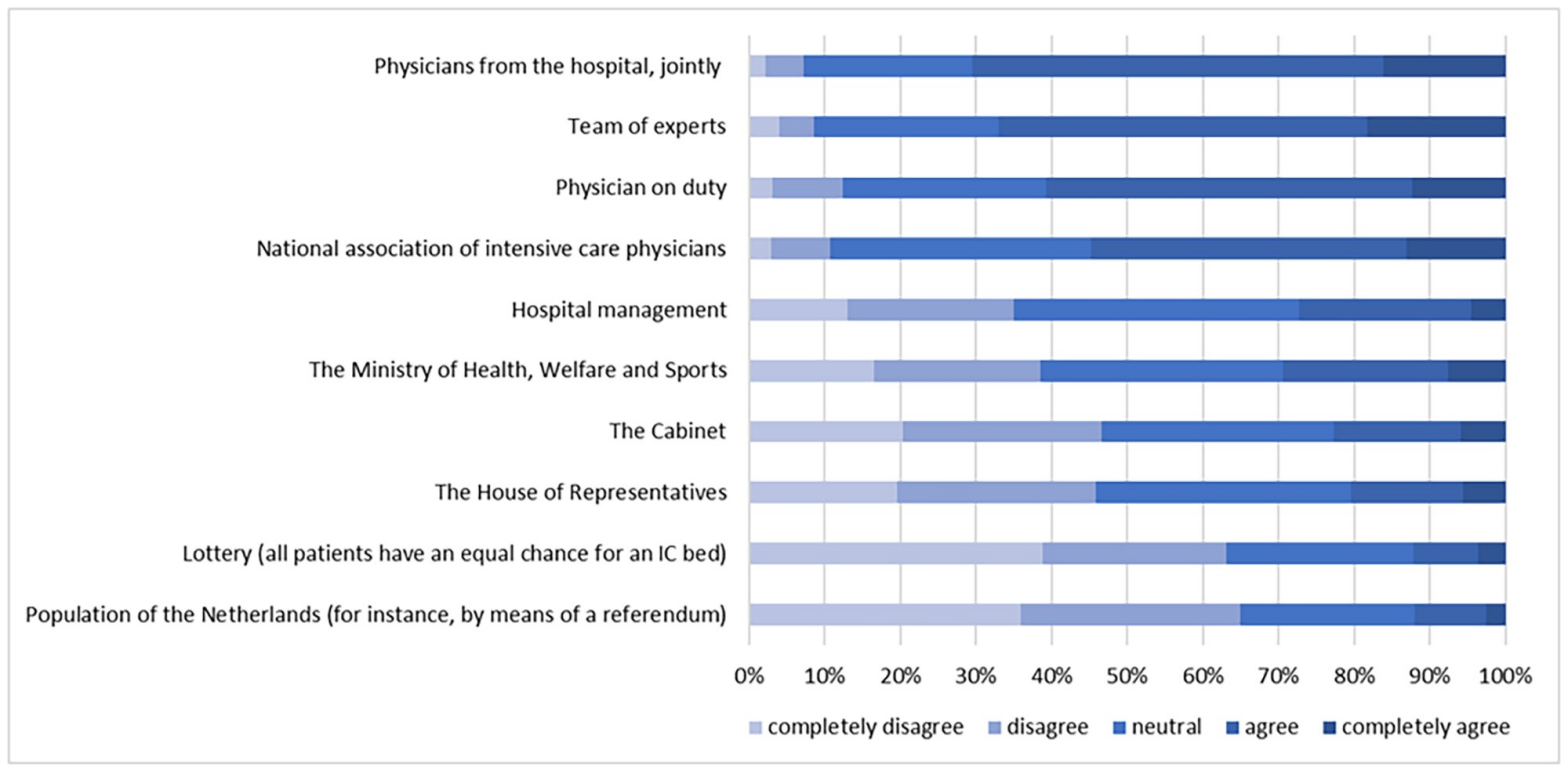
Support for decision-making categories, ranked by agreement.

Fig 1 shows that large majorities of between 55% and 70% of the respondents completely agreed that physicians from the hospital making a joint decision, the physician on duty, the national association of intensive care physicians, or a team of experts should play a role in developing guidelines for the allocation of ICU beds. Much smaller proportions of between 20% and 30% thought the Ministry of Health, Welfare and Sports, the Cabinet, the House of Representatives, or hospital management should play a role. When aggregating these decision-makers into the categories defined earlier, the majority of the sample were in favor of a role for *physicians* (55%) or an *expert committee* (51%), while about 18% considered *government* to be an appropriate decision-maker. Only 12% of the respondents were in favor of a role for *the public*, and only 12% were in favor of a *lottery* (see Supporting information, S3 Table).

Overall, about one-third of the respondents (34.3%) had a clear preference for a single decision-maker category, while 43.9% were in favor of shared responsibility between two or more of the proposed decision-maker categories (see S3 Table and S1 Fig in the Supporting information). Approximately one out of five respondents (21.8%) did not support a role for any of the five decision-maker categories, as indicated by an average score of less than 4 for all categories. About half of this group (114 respondents; 11.2% of the total sample) even had an average score of less than 3 for all categories. These results could be interpreted as protest responses, because the majority of these respondents (74%) did not provide an alternative suggestion for who should be involved in decisions about the allocation of ICU beds. Moreover, many of these respondents expressed dissatisfaction with the government and the shortage of ICU beds in a wealthy country such as the Netherlands (example: “*The shortage of ICU beds is simply the result of all the cutbacks implemented by the current and previous governments*”; resp100). Some characterized having anyone play a role in allocating ICU beds as inhumane and expressed relief that they themselves did not bear the responsibility for such decisions (example: “*Everything should be done to avoid having physicians and patients ending up in these sorts of situations*”; resp31). This “protest group” had a slightly lower mean age than the rest of the sample (45 years versus 48 years) and was less educated. No differences were found regarding sex or employment status.

The results of the models investigating the associations between the preferences regarding who should be involved in decisions about the allocation of ICU beds and the demographic characteristics and opinions of respondents are presented in Table 4. We found that people aged 60–77 years were 16 percentage points more likely to be in favor of a role for physicians, while younger people were more likely to be in favor of a role for government or a lottery. Female respondents were more likely than male respondents to be in favor of a role for an expert committee. Compared to respondents with a low level of education, respondents with a medium level of education were 9 percentage points less likely to be in favor of an expert committee, and those with a high level of education were 7 percentage points less likely to be in favor of a role for the public. Being employed increased the likelihood of being in favor of a role for the government or the public. In addition, compared to the respondents who considered the government’s response to the COVID-19 pandemic (highly) insufficient, those who believed it was (highly) exaggerated were more likely to be in favor of a role for the public or a lottery, and less in favor of an expert committee. Compared to respondents who considered the measures the government took in response to the pandemic (highly) ineffective, those who thought they were (highly) effective were more likely to be in favor of a role for physicians, an expert committee, and the government. Finally, stockpiling during the initial stages of the COVID-19 pandemic was positively associated with a role for the public or a lottery, and to a smaller extent also with a role for the government.

**Table 4:** Probit regressions preferences for decision-makers; average marginal effect (95% confidence interval).

	Who should decide who gets an ICU bed?									
	Physicians		Expert committees		Government		The public		Lottery	
Age										
18–34	ref		Ref		ref		Ref		ref	
35–59	0.12**	(.03; .19)	0.03	(-.05; .11)	-0.04	(-.10; .02)	-0.01	(-.06; .03)	-0.07*	(-.12; -.01)
60–77	0.16**	(.06; .25)	-0.06	(-.15; .03)	-0.08*	(-.15; -.00)	-0.01	(-.07; .04)	-0.07*	(-.13; .00)
Sex										
Male	Ref		Ref		Ref		Ref		Ref	
Female	0.05	(-.01; .11)	0.07*	(.01; .13)	-0.04	(-.09; .00)	-0.04	(-.07; .00)	-0.03	(-.07; .00)
Education level										
Low	ref		Ref		Ref		Ref		Ref	
Middle	0.06	(-.02; .13)	-0.09*	(-.17; -.01)	-0.05	(-.11; .01)	-0.02	(-.07; .03)	0.00	(-.04; .05)
High	0.01	(-.08; .09)	0.00	(-.08; .08)	-0.06	(-.12; .00)	-0.07*	(-.11; -.01)	-0.04	(-.09; .01)
Employed										
No	ref		Ref		ref		Ref		Ref	
Yes	-0.05	(-.12; .02)	-0.04	(-.11; .03)	0.06*	(.00; .10)	0.07**	(.02; .10)	0.02	(-.02; .06)
Government response										
(Highly) Insufficient	ref		Ref		ref		Ref		Ref	
Appropriate	-0.02	(-.09; .05)	-0.07	(-.14; .00)	0.01	(-.04; .06)	-0.00	(-.04; .04)	0.02	(-.02; .05)
(Highly) Exaggerated	0.01	(-.11; .12)	-0.15*	(-.27; -.03)	0.05	(-.05; .13)	0.12**	(.03; .21)	0.09*	(.00; .17)
Government measures										
(Highly) Ineffective	Ref		Ref		Ref		Ref		Ref	
Neutral	-0.00	(-.09; .05)	-0.00	(-.10; .10)	0.05	(-.01; .11)	-0.02	(-.09; 0.4)	-0.00	(-.06; .06)
(Highly) Effective	0.14**	(.04; .25)	0.19***	(.09; .29)	0.10**	(.03; .16)	-0.05	(-.11; .01)	-0.00	(-.06; .05)
Stockpiling										
No	Ref		Ref		ref		Ref		Ref	
Yes	-0.01	(-.07; .06)	0.06	(-.00; .13)	0.05*	(.00; .11)	0.10***	(.05; .15)	0.10***	(.04; .14)
McFadden’s R2	0.034		0.041		0.034		0.093		0.066	
Observations	1019		1019		1019		1019		1019	

* *p* < 0.05,

** *p* < 0.01,

*** *p* < 0.001.

### Which criteria should be considered in deciding on the allocation of ICU beds?

**Fig 2:**
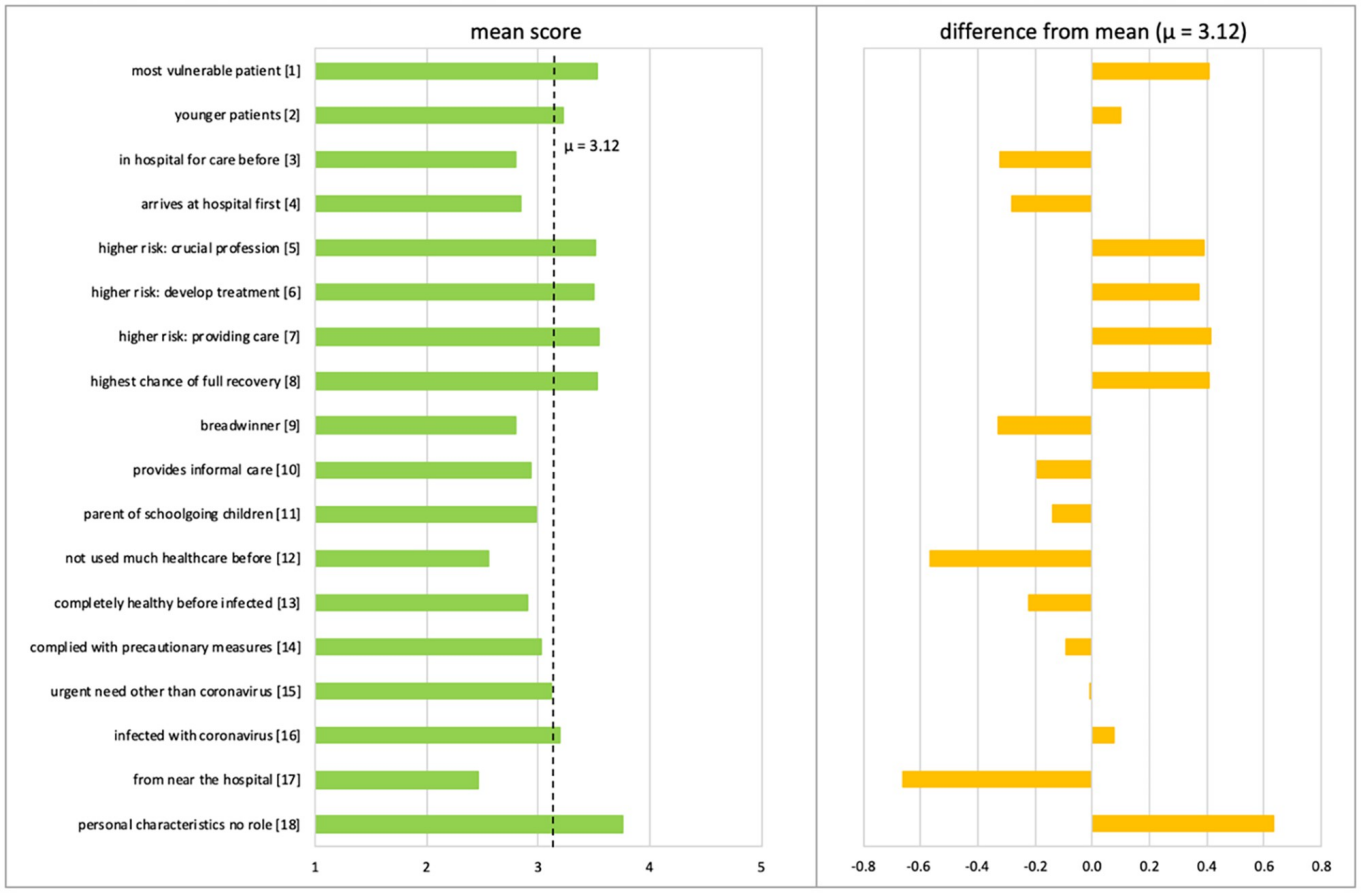
Preferences for decision-making criteria for the allocation of ICU beds (mean score and difference from overall mean).

The left panel of Fig 2 shows the mean score for each of the 18 criteria presented to respondents. The overall mean score was 3.12 (on a scale ranging from 1 to 5). The right panel of Fig 2 shows the difference between the mean score for each criterion and the overall mean score.

The highest mean score (3.8) was observed for the criterion [18] stating that personal characteristics should play no role in the allocation of ICU beds. This criterion representing the equality of patients was followed by criteria [1] and [8], favoring patients who are vulnerable or have the highest chance of full recovery, and criteria [5,6], and [7], favoring those with higher risks related to working in a crucial profession, caring for infected patients, or working on development of a vaccine or treatment. Prior healthcare use [12] and the hospital-related criteria [3] and [17], which received the lowest mean scores, were thus least preferred for consideration in guidelines for the allocation of ICU beds. Compliance with the safety measures advised by the government [14], which involves notions of own responsibility and culpability, also received a lower-than-average score. Preference for prioritizing patients infected with the coronavirus [16] was only slightly higher than for patients with other urgent needs for an ICU bed [15].

We further investigated how the six allocation criteria that either came out as the most relevant in our data ([1,5,8] and [18]) or were most prolific in the public debate in the Netherlands at the time of analysis ([2] and [14]) associated with respondents’ demographic characteristics and preferences for decision-maker categories, see Table 5 for model estimations. We found that age, education level, and employment status affected the likelihood of being in favor of certain criteria. For example, people aged 35–77 were more likely than young people to support prioritizing based on vulnerability [1] and less likely to support prioritizing based on age [2]. The oldest age group was less likely to support prioritizing based on patients’ personal characteristics [18], while people aged 35–59 were less likely to support the culpability criterion [14]. More highly educated people were more likely to be in favor of prioritizing based on the capacity to benefit [8]. Employed people were less likely than unemployed people to be in favor of the vulnerability criterion [1] and more likely to be in favor of the culpability criterion [14] and the capacity to benefit [8].

**Table 5:** Probit regressions decision-making criteria; average marginal effect (95% confidence interval).

Demographic characteristics	What should determine the allocation of ICU beds?											
	Vulnerability [1]		Age [2]		Crucial profession [5]		Chance of full recovery [8]		Compliance with measures [14]		Personal characteristics no role [18]	
Age												
18–34	ref		ref		ref		Ref		ref		ref	
35–59	0.08*	(-.00; .17)	-0.12**	(-.20; -.04)	-0.05	(-.13; .03)	-0.04	(-.12; .04)	-0.09*	(-.16; .01)	-0.03	(-.11; .05)
60–77	0.11*	(-.01; .20)	-0.13**	(-.22; -.04)	0.05	(-.05; .14)	0.03	(-.07; .13)	-0.08	(-.16; .01)	0.13**	(.04; .22)
Sex												
Male	ref		Ref		Ref		Ref		Ref		Ref	
Female	-0.02	(-.08; .04)	0.03	(-.03; .09)	-0.01	(-.07; .06)	0.02	(-.04; .08)	-0.01	(-.06; .05)	0.05	(-.01; .12)
Education level												
Low	ref		Ref		Ref		Ref		Ref		Ref	
Middle	-0.04	(-.12; .04)	-0.04	(-.11; .04)	-0.06	(-.14; .02)	0.04	(-.04; .12)	-0.07	(-.15; .00)	-0.05	(-.13; .03)
High	-0.06	(-.14; .03)	0.05	(-.03; .14)	-0.02	(-.11; .06)	0.13**	(.04; .21)	-0.03	(-.11; .04)	-0.04	(-.12; .05)
Employed												
No	ref		Ref		Ref		Ref		Ref		Ref	
Yes	-0.09*	(-.16; -.01)	0.02	(-.05; .09)	-0.01	(-.08; .06)	0.08*	(.00; .15)	0.07*	(.01; .14)	0.01	(-.06; .08)
Physicians												
Neutral/disagree	ref		Ref		Ref		Ref		Ref		Ref	
Agree	0.16***	(.09; .22)	0.04	(-.03; .10)	0.12***	(.05; .19)	0.15***	(.08; .21)	-0.01	(-.07; .05)	0.16***	(.10; .23)
Expert committee												
Neutral/disagree	ref		Ref		Ref		Ref		Ref		Ref	
Agree	0.07*	(.00; .14)	0.11**	(.04; .17)	0.18***	(.11; .24)	0.13***	(.07; .20)	0.13***	(.07; .19)	0.06	(-.01; .12)
Government												
Neutral/disagree	ref		Ref		Ref		Ref		Ref		Ref	
Agree	0.04	(-.05; .13)	0.12*	(.03; .21)	0.09*	(.00; .19)	0.14**	(.05; .23)	0.11*	(.02; .19)	-0.03	(-.13; .06)
The public												
Neutral/disagree	Ref		Ref		Ref		Ref		Ref		Ref	
Agree	0.08	(-.02; .20)	0.09	(-.02; .20)	0.03	(-.08; .15)	-0.09	(-.21; .02)	0.10	(-.00; .20)	-0.00	(-.11; .11)
Lottery												
Neutral/disagree	ref		Ref		Ref		ref		Ref		Ref	
Agree	0.10	(-.00; .20)	0.04	(-.06; .15)	0.20***	(.11; .30)	0.09	(-.02; .19)	0.15**	(.04; .25)	0.15**	(.05; .24)
McFadden’s R2	0.054		0.053		0.076		0.061		0.081		0.051	
Observations	1019		1019		1019		1019		1019		1019	

* *p* < 0.05,

** *p* < 0.01,

*** *p* < 0.001.

Moreover, people in favor of a role for physicians were more likely to support the criteria related to vulnerability [1], work-related risk [5], and capacity to benefit [8] but against discrimination based on personal characteristics [18]. People in favor of a role for an expert committee were more likely to support all of the allocation criteria other than the one specifying that personal characteristics should play no role [18]. People in favor of a role for the government were likely to support the age [2] and crucial profession [5] criteria, which the government in the Netherlands actually does, but also the chance of full recovery [8] and culpability [14] criteria, which it does not. As one would perhaps expect, people in favor of a role for a lottery were against discrimination based on personal characteristics [18], yet they showed support for the crucial profession [5] and culpability [14] criteria.

## Discussion

The data for this study were collected at the end of the first quarter of 2020, when the first wave of the COVID-19 outbreak in the Netherlands was nearing its peak. Hence, this study took place against the backdrop of public and political debates about the increasing pressure the pandemic placed on the healthcare system. In this context, we wanted to investigate societal preferences regarding the allocation of scarce ICU beds during a health crisis. We assessed the societal preferences for various types of decision-makers being involved in the development of guidelines for the allocation of ICU beds and the rationing criteria that should be considered in this allocation process.

The results of our study suggest that the majority of the sample believed that physicians or an expert committee should be involved in developing guidelines for the allocation of scarce ICU beds during a health crisis. The preferred allocation criteria for guiding these decisions mostly related to the health and risk profiles of patients in need of an ICU bed. Priority for the most vulnerable patients and those with the highest chance of full recovery was supported, as well as priority for those with a higher work-related risk of becoming infected. Interestingly, it was generally preferred that personal characteristics should not to play a role, and priority for younger patients was only weakly supported. The age criterion has been criticized more generally before, both by experts and in the public debate, as connected with ageism or even racism [7]. One of the arguments against an age-related criterion, which may also have played a role in our study, is that research shows significant differences between biological and chronological age [23]. The weak support for using age as a decision criterion seems to stand in contrast with international guidelines, where age appears to be a leading criterion for prioritizing patients when there is a shortage of ICU beds [6,18]. However, other criteria considered important in our study are in part age-related, for example, vulnerability and the chance of full recovery. Based on our findings, we anticipate that guidelines based on age may be met with opposition from the public, although the clinical reasoning for using age as a criterion may not be so different from the priorities of the public. Presenting respondents separately with the different criteria previously identified in the literature enabled us to disentangle the various criteria that may otherwise be conflated as an age-related criterion. However, we recognize that there is not a single value alone that is able to determine which patients should be prioritized. Rather a multivalue ethical framework should be applied [24]. As suggested also by others, a utilitarian perspective (e.g. greatest benefit), individual patient preferences, social contexts, and operability should be included in the decision-making process [25].

We found that respondents who were 35 years and older were more positive about a role for physicians in developing allocation guidelines, while respondents who were younger than 35 more strongly supported a lottery. We also found that people who were positive about how the Netherlands’ government is handling the pandemic, that is, who were satisfied with the government’s response to the COVID-19 outbreak and considered the measures taken by the government to be effective, were more likely to be in favor of a role for the government. They were also more positive about a role for an expert committee, which, considering the government’s strong reliance on such a committee (called the “outbreak management team”) in the development of their policies for handling the pandemic, seems to make sense. Not surprisingly, people who were positive about how the government was handling the current health crisis also tended to prefer a role for government in the development of guidelines for the allocation of ICU beds, and people who considered the government response as exaggerated were more likely to be in favor of a role for the public or a lottery. Finally, people who experienced more uncertainty in relation to the pandemic, as revealed by self-reported stockpiling behavior, showed stronger support for a role for the government, the public, and a lottery, but not for physicians or an expert committee. One possible interpretation of this finding is that people who experienced more uncertainty generally do not trust or understand or feel insufficiently represented in the advice of experts as much as others do and would therefore like to shift influence away from experts and make the government more accountable for their decisions, or, alternatively, leave it to the public or a lottery.

Approximately one out of every nine respondents was neutral or (strongly) disagreed with a role for any of the proposed decision-makers, but also did not provide alternative suggestions. In the open follow-up question, some of these respondents expressed the belief that rationing ICU beds is inhumane, with the government to blame for the capacity shortage, and that they were glad they were not—and also did not want to be—responsible for such difficult choices. This “protest response” could also be interpreted as *decision avoidance*. When respondents perceive themselves to be personally responsible if they state being in favor of something, they may more likely anticipate regret about the possible outcomes of their choices and hence may prefer not to choose [26]. Avoidance of a decision, in particular deferral, is more likely among decision-makers who hope to postpone or escape the responsibility of making a decision [26].

Societal preferences for healthcare priority setting have previously been assessed in the Netherlands, although under ordinary circumstances [10,11,20,27]. Across these studies, an egalitarian view with respect to decision-making in healthcare was found to be most common, emphasizing the importance of equal opportunities and access to healthcare services for those in need of care. This is in line with our finding that vulnerability should play a role in the prioritization of patients for ICU beds, while personal characteristics, including age, should not. These previous studies also found that the effectiveness of the care and the quality of life after treatment are considered important by members of the public in the Netherlands, which seems to be consistent with the strong support for prioritizing those with a chance of full recovery in our study. In the context of limited ICU bed capacity, preference for those who would benefit most relates to a concern for the efficiency of healthcare. Hence, also in the context of a health crisis, people seem to trade off concerns about equity and about efficiency. These trade-offs differ for different people; in our study, more highly educated people in particular seemed more in favor of considering efficiency in the allocation of ICU beds.

Some limitations of our study need to be acknowledged. First, we collected our data by means of an online survey, and the answers to certain questions may be sensitive to a social desirability bias [28]. Second, our data collection took place at the start of the COVID-19 outbreak. Although the shortage of ICU beds was a realistic threat for the country’s healthcare system at the time of data collection, it did not materialize because of intensive investments in capacity and deferral of patients to a neighboring country, Germany. In addition, the data collection took place in the early days of the pandemic, and since then much has changed regarding the patient flow within and between hospitals and the ICU treatment capacities and efficiency. Therefore, public opinion may also have changed in the interim. Repeating this study today, a year later, while the Netherlands is facing a third wave of COVID-19 infections, could generate additional insights about societal preferences for rationing healthcare during a health crisis. Some criteria might have become less or more relevant in the eye of the public. One could hypothesize that after months of experience with the social and economic consequences of lockdown measures, and now that people are better informed and more aware about the behavioral component in preventing contamination, the culpability criterion may have gained popularity. Finally, although our data were collected from a sample that was intended to be representative of the adult population of the Netherlands (in terms of age, sex, and level of education), caution is required in generalizing our findings. The sample ended up being slightly older and more highly educated than the reference population, and it cannot be ruled out that certain subgroups of the overall population were less likely to accept the invitation to participate or to finish completing the survey. Moreover, although the COVID-19 pandemic is an international concern, generalization of our findings beyond the Netherlands is limited by differences between countries in the organization and capacity of their healthcare systems, the measures taken by governments to contain and mitigate the coronavirus, and more general value orientations in the population (such as equality and solidarity).

## Conclusions

In conclusion, it appears that during a health crisis, the public attaches the most value to rationing criteria that are related to the health status and prospects of patients and to their risk profiles and not to personal characteristics such as their age. The majority of our sample shared the opinion that physicians and experts should be responsible for the development of guidelines for the allocation of scarce ICU beds. The considerable size of the “protest group” that did not support any of the decision-makers or did not want to bear any responsibility for this type of decision signals that any healthcare rationing decision in the context of a health crisis may face considerable opposition. Hence, policy makers should devote extra attention to disseminating information regarding the importance of rationing criteria in the context of healthcare. Moreover, allocation guidelines that involve criteria related to the health and risk profiles of patients as well as those that favor patients that have the highest chances of full recovery are likely to receive the most support from the public.

## Supporting information

S1 Fig Number of decision-maker categories preferred. (DOCX)

S1 Table Decision-makers; spearman correlation matrix agreement scores. (DOCX)

S2 Table COVID-19 related questions. (DOCX)

S3 Table Preferences for who should be involved in the allocation of ICU beds (N *=* 1,019). (DOCX)

## Abbreviations

ICU: Intensive Care Unit
SIAARTI: Societa’ Italiana Anestesia Analgesia Rianimazione e Terapia Intensiva
WHO: World Health Organization
