# Supplementary figures and images for: How should ICU beds be allocated during a crisis? Evidence from the COVID-19 pandemic
Source: PLoS One. 2022 Aug 10;17(8):e0270996. doi: 10.1371/journal.pone.0270996 (PMC9365136; doi:10.1371/journal.pone.0270996)

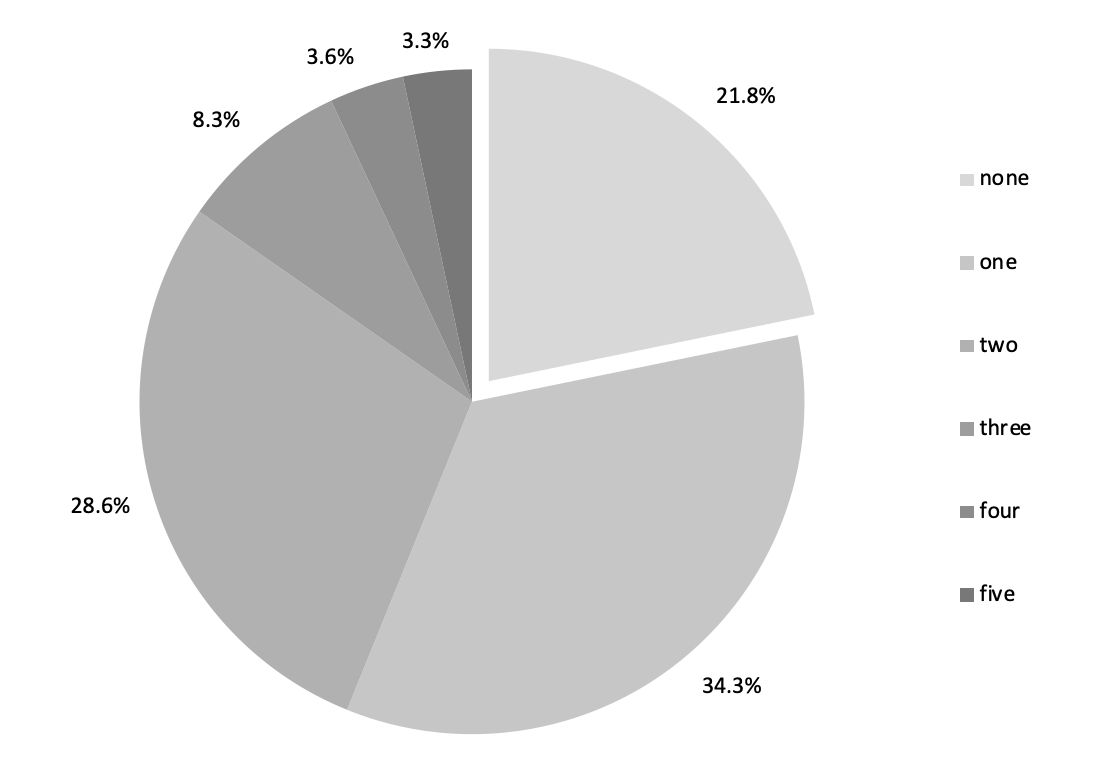

Supplement: S1 Fig — (DOCX) [file pone.0270996.s001.docx]
