# Supplementary material for: How should ICU beds be allocated during a crisis? Evidence from the COVID-19 pandemic
Source: PLoS One. 2022 Aug 10;17(8):e0270996. doi: 10.1371/journal.pone.0270996 (PMC9365136; doi:10.1371/journal.pone.0270996)
Supplement: S1 Table — (DOCX) [file pone.0270996.s002.docx]

|  |  | 1. | 2. | 3. | 4. | 5. | 6. | 7. | 8. | 9. |
| --- | --- | --- | --- | --- | --- | --- | --- | --- | --- | --- |
| 1. | Physician on duty | 1.00 |  |  |  |  |  |  |  |  |
| 2. | Physicians from the hospital jointly | 0.56 | 1.00 |  |  |  |  |  |  |  |
| 3. | National association of intensive care physicians | 0.26 | 0.44 | 1.00 |  |  |  |  |  |  |
| 4. | Population of the Netherlands (for instance by means of a referendum) | -0.08 | -0.12 | 0.07 | 1.00 |  |  |  |  |  |
| 5. | Hospital management | 0.02 | 0.12 | 0.25 | 0.43 | 1.00 |  |  |  |  |
| 6. | The House of Representatives | -0.10 | -0.07 | 0.22 | 0.50 | 0.52 | 1.00 |  |  |  |
| 7. | The Cabinet | -0.07 | -0.02 | 0.24 | 0.46 | 0.49 | 0.89 | 1.00 |  |  |
| 8. | The Ministry of Health, Welfare and Sports | -0.06 | 0.02 | 0.31 | 0.40 | 0.49 | 0.75 | 0.80 | 1.00 |  |
| 9. | Team of experts | 0.19 | 0.36 | 0.47 | -0.03 | 0.24 | 0.18 | 0.21 | 0.31 | 1.00 |
| 10. | Lottery (all patients have an equal chance to an ICU bed) | -0.13 | -0.11 | -0.02 | 0.48 | 0.29 | 0.31 | 0.29 | 0.24 | -0.02 |
