# Supplementary material for: How should ICU beds be allocated during a crisis? Evidence from the COVID-19 pandemic
Source: PLoS One. 2022 Aug 10;17(8):e0270996. doi: 10.1371/journal.pone.0270996 (PMC9365136; doi:10.1371/journal.pone.0270996)
Supplement: S2 Table — (DOCX) [file pone.0270996.s003.docx]

| **Question** | **Response categories** |
| --- | --- |
| What do you think of the response of the government to the COVID-19 outbreak in the Netherlands? | highly exaggerated; exaggerated; appropriate; insufficient; highly insufficient |
| How effective do you consider current measures taken by the government in order to slow down the spread of the COVID-19 virus? | very ineffective; ineffective; neutral; effective; very effective |
| During the last three days, have you bought more of certain things than you would do in normal circumstances? | yes, of many things; yes, of some things; no |
