# Supplementary material for: How should ICU beds be allocated during a crisis? Evidence from the COVID-19 pandemic
Source: PLoS One. 2022 Aug 10;17(8):e0270996. doi: 10.1371/journal.pone.0270996 (PMC9365136; doi:10.1371/journal.pone.0270996)
Supplement: S3 Table — (DOCX) [file pone.0270996.s004.docx]

| **Decision-maker category** | **Preferred** |  | **Preferred as only decision-maker** |  | **Also a role for…** | | | | |
| --- | --- | --- | --- | --- | --- | --- | --- | --- | --- |
|  |  |  |  |  | **Physicians** | **Expert committee** | **Government** | **The public** | **Lottery** |
|  | **N (%)** |  | **N (% of category)** |  | **N (% of category)** | | | | |
| Physicians | 560 (55.0) |  | 181 (32.7) |  | - | 357 (63.8) | 113 (20.2) | 78 (13.9) | 67 (12.0) |
| Expert committees | 520 (51.0) |  | 104 (20.0) |  | 357 (68.7) | - | 151 (29.0) | 85 (16.3) | 79 (15.2) |
| Government | 179 (17.6) |  | 19 (10.6) |  | 113 (63.1) | 151 (84.4) | - | 65 (36.3) | 52 (29.1) |
| The public | 122 (12.0) |  | 17 (13.9) |  | 78 (63.9) | 85 (69.7) | 65 (53.3) | - | 61 (50.0) |
| Lottery | 124 (12.2) |  | 16 (21.8) |  | 67 (54.0) | 79 (63.7) | 52 (41.9) | 61 (49.2) | - |
| None | 222 (21.8) |  | - |  | - | - | - | - | - |
